# Psychological resources in adolescence and the association with labour market participation in early adulthood: a prospective cohort study

**DOI:** 10.1186/s12889-020-08531-w

**Published:** 2020-03-24

**Authors:** Jacob Devantie Jensen, Johan Hviid Andersen, Trine Nøhr Winding

**Affiliations:** 1 Ramboll Management Consulting, Stakeholder Intelligence, Olof Palmes Allé 22, 8200 Aarhus, Denmark; 2 Department of Occupational Medicine, Danish Ramazzini Centre, University Research Clinic, Regional Hospital West Jutland, Gl. Landevej 61, 7400 Herning, Denmark

**Keywords:** Labour market participation, Adolescents, Personal mastery, Self-esteem, Sense of coherence

## Abstract

**Background:**

Young adults at the age of 25–29 in Denmark have the highest unemployment rate and are at higher risk of labour market marginalization. Exclusion from the labour market may have negative individual consequences on mental and physical health and can lead to increasing societal expenditures due to social benefits. It is important to understand what factors determine or protect against early labour market marginalization. The aim of the present study was to investigate the association between psychological resources in adolescence and labour market participation in early adulthood, and whether the associations differed by gender.

**Methods:**

This prospective cohort study used questionnaire data collected through the West Jutland Cohort study in 2004 and 2007. The study population (*N* = 2982) consisted of people born in 1989 and living in the county of Ringkjoebing at baseline in 2004. Outcome was dichotomized as +/− 12 months of passive labour market participation during the age of 25–29. Psychological resources were measured as self-esteem, sense of coherence and mastery. Logistic regression analyses were performed to investigate the associations between psychological resources and labour market participation.

**Results:**

Results indicated associations between high levels of mastery or sense of coherence in adolescence and high labour market participation in early adulthood. The strongest associations were observed for females with a medium (OR: 1.9, 95% CI: 1.3–2.8) or high level (OR: 1.6, 95% CI: 1.0–2.4) of mastery or a high level of sense of coherence (OR: 1.6 95% CI: 1.0–2.4) at age 15 and for males with a medium (OR: 2.7, 95% CI: 1.5–3.8) or high (OR: 1.9, 95% CI: 1.1–3.5) level of mastery or a high level of sense of coherence (OR: 1.7, 95% CI: 0.9–3.1) at age 18.

**Conclusion:**

The results of the present study indicate associations between a high level of sense of coherence or mastery in adolescence and high labour market participation in early adulthood in a Danish context. Psychological resources seemed to play a bigger role for females in early adolescence compared to males, for whom a larger impact was seen in late adolescence.

## Background

In February 2019 the general unemployment rate in Denmark was 3.7%; however it was 7% for 25–29-year-olds [1]. Labour market marginalization among young adults is of rising concern due to the related negative health consequences [2]. Young adults outside the labour market are specifically vulnerable to general health problems [3] and show a higher risk of psychological vulnerability and distress and feel affected by insecurities about their own capabilities [4]. Stability of attachment to the labour force during the transitional period into working life is of great importance, as it improves the future job prospects and avoids the dependency on social benefits [5].

Young people from the Nordic countries who experience unemployment have a greater risk of experiencing unemployment across the life course, regardless of their individual characteristics or background [6, 7]. Furthermore, there seems to be an increased risk of a marginalized income later in life in people being economically inactive during young adulthood [8]. However, the long term effects of youth unemployment may depend on the individuals ability to cope with their circumstances [9]. The entrance into the labour market in early adulthood is therefore an important part of the general life course development and it is important to study the determinants of facilitating labour market participation (LMP) in early adulthood.

Previous studies show clear associations between socioeconomic status such as low income or educational level in the family, psychiatric disorders, the experience of negative life events, somatic complaints during adolescence and the risk of an unstable entrance into the labour market in early adulthood [10–14]. Furthermore, indications point towards mental health and psychological vulnerability affecting the early work environment [15]. Many studies focus on the effect of unemployment on mental health factors. However, few studies focus on the resilient perspective of psychological factors as resources in facilitating LMP.

Adolescence is a key-developmental stage of both physical, neurological, psychological and social changes and have immense impact on later health outcomes leaving some individuals more vulnerable than others [16]. Psychological resources appear to develop across the entire life span, with both self-esteem [17, 18], personal mastery [19] and sense of coherence [20, 21] being at their lowest point during adolescence. Furthermore, gender seems to be a contributing factor in the level of self-esteem, personal mastery and sense of coherence in primarily Western and Nordic countries. Males generally show a higher level of the respective psychological resources independent of age [19, 20, 22]. A previous study on the same cohort found that males reporting somatic symptoms in late adolescence appeared to be more vulnerable to future reduced labour market participation compared to the females [11]. However, it is still not known if self-esteem, personal mastery or sense of coherence affect later labour market participation. Psychological resources are important factors of general health and can act as protective determinants during life course transitions [23]. Psychological resources such as self-esteem and sense of coherence (SOC) have shown to be facilitating factors of entering the labour market. Furthermore, they are also affected negatively from staying excluded from the labour market. A previous study indicated that the vulnerability of unemployed depends on their individual ability to cope with unemployment, which refers to the individual’s psychological resources [9]. These indications point to a more bidirectional relationship between LMP and the respective psychological resources of the present study, as they reciprocally affect each other. As described above, labour market marginalization on one hand affects psychological resources negatively, which makes it harder to enter the labour market, as they also act as facilitating factors. On the other hand, unemployment affects psychological resources negatively. This enforces the motivation and relevance of studying the relationship more carefully. However, previous studies are primarily based on cross-sectional data and relatively small datasets [17, 24–26]. Research suggests an association between a lower level of sense of coherence and the risk of receiving social benefits in young adulthood [27]. Adolescents with a low level of self-esteem have shown to attain lower levels of education and are less likely to be employed 14-years after leaving high-school [28]. People with higher self-esteem seem to handle negative life events better [29], and show greater success in the initial job search [30]. A sense of mastery reduces psychological distress and act as a protective agent against stressful life events. In addition, a sense of mastery seems to be a mediating factor between the role of social class and economic hardship and health later in life [31–33]. A high level of SOC seems to be related to positive perceived health and show strong inverse associations with psychological symptoms like depression and anxiety [34]. The association between self-esteem and depression has been extensively studied, showing a strong association between low self-esteem and depression; however, the causal link is broadly debated [35]. Research in regard to mastery has focused more on the mediating role of mastery on depression, and higher levels of mastery indicate lower risk of depression [36]. People from lower levels of socioeconomic status, most strongly related to income and education, generally appear to have lower levels of self-esteem, sense of coherence and mastery [19, 37, 38].

The existing knowledge of the link between psychological resources of self-esteem, mastery and sense of coherence in adolescence and LMP in early adulthood is sparse. Given the nature and existing knowledge of the harmonious facilitating capabilities of these psychological resources, they ensure potential in advancing LMP in early adulthood. The present study seeks to investigate the associations between self-esteem, sense of coherence or mastery at age 15 and 18 and LMP in early adulthood at age 25–29. Furthermore, the study aims to investigate a potential gender difference in the associations between these psychological resources and LMP.

## Methods

### Study population

The study population was derived from the West Jutland Cohort study, which is a project focusing on studying and exploring different aspects of inequalities and social disparities on general health and well-being in a life course perspective [39]. The West Jutland Cohort is a follow-up study of 3681 individuals, who were born in 1989 and living in the western part of Jutland, Denmark in 2004 [11, 40]. The recruitment took place at the schools within the county of Ringkjoebing, subsequently the children who were not at school received the questionnaire by mail. From the original source population (*n* = 3681), 3054 (83%) adolescents (age 15) participated in filling out the baseline questionnaire in 2004 [40]. A follow-up questionnaire was distributed by postal service and mail in 2007 when the adolescents were 18 years old. The follow-up resulted in 2181 participants (71% of the initial study population). However, since the follow-up questionnaire was distributed to all 3681 individuals from the source population, 219 new respondents participated. The final study population for the present study included baseline participants with questionnaire information on psychological resources at age 15 or 15 and 18 with register information on LMP in the period between the age of 25–29 (*n* = 2982). The number of responders at age 15 (*n* = 2982) was reduced due to loss to follow-up by (*n* = 809), leaving the following number of responders at age 18 (*n* = 2173). The broad research perspective of the West Jutland Cohort study, together with the longitudinal data, makes it suitable for investigating how different psychological resources are associated with LMP and at the same time adjust for potential confounders.

### Outcome

The information of the outcome for the present study was obtained from the Danish Register for Evaluation and Marginalization (DREAM) [41]. The DREAM register includes information on social benefits such as unemployment benefits, sickness benefits and disability benefits on a weekly basis on all Danish citizens. Information about social benefits for a 204-week period was collected from 1st of January 2015 to 2nd of December 2018, when the participants were 25–29 years old. For all participants, weeks not receiving any benefits at all, or receiving maternity leave benefits, state educational benefits or supplementary unemployment benefits were classified as “active LMP”. On the contrary weeks of labour market exclusion receiving all other kinds of social benefits were classified as “passive LMP”. Based on the total number of weeks with passive LMP during the 4 years period, participants were dichotomized into ‘Low LMP’ if they had received ≥12 months of passive LMP or ‘High LMP’ if they had received < 12 months of passive LMP. The DREAM-register provided outcome information on all participants from 2004 and 2007.

The register information was merged with questionnaire data by the Danish CPR-number which every Danish inhabitant possesses [42].

### Exposure variables

In the present study, psychological resources were measured as the theoretical concepts of self-esteem, mastery and sense of coherence. Information on these resources was obtained through questionnaire data in 2004 (age 15) and 2007 (age 18). The three variables were collected through subscales of earlier validated questionnaires; sense of coherence (Antonovsky’s sense of coherence scale) [43], personal mastery (Pearlin & Schooler) [44] and self-esteem (Rosenberg’s Self-esteem scale) [45]. The full scales were not included for sense of coherence and self-esteem. The included scales are described in Table 1.

**Table 1:** Exposure items from the included scales to measure psychological resources

Items	Scale
**Sense of coherence (Meaningfulness)**	Total score sums between 4 and 20
1. What do you think of the things you do every day?	“Very boring” (1) – “Very exciting” (5)
2. How often do you do things that you find meaningful?	“Never” (1) – “Very often” (5)
3. How often do you feel, that you don’t care about what goes on around you?	“Very often” (1) – “Never” (5)
4. How often do you have the feeling that there is little meaning in the things you do in your daily life?	“Very often” (1) – “Never” (5)
**Personal mastery**	Total score sums between 7 and 28
1. I have little control of the things that happen to me.	“Strongly disagree” (1) – “Strongly agree” (4)
2. There is really no way I can solve some of the problems I have	“Strongly disagree” (1) – “Strongly agree” (4)
3. There is little I can do to change many of the important things in my life	“Strongly disagree” (1) – “Strongly agree” (4)
4. I often feel helpless in dealing with problems of life	“Strongly disagree” (1) – “Strongly agree” (4)
5. Sometimes I feel that I’m being pushed around in life	“Strongly disagree” (1) – “Strongly agree” (4)
6. What happens in the future mostly depends on me	“Strongly disagree” (1) – “Strongly agree” (4)
7. I can do just about anything I really set my mind to do	“Strongly disagree” (1) – “Strongly agree” (4)
**Self-esteem**	Total score sums between 6 and 24
1. I feel that I have a number of good qualities	“Strongly disagree” (1) – “Strongly agree” (4)
2. I feel that I’m a person of worth at least equal to others	“Strongly disagree” (1) – “Strongly agree” (4)
3. I am able to do things as well as most other people	“Strongly disagree” (1) – “Strongly agree” (4)
4. I take a positive attitude toward myself	“Strongly disagree” (1) – “Strongly agree” (4)
5. On a whole, I am satisfied with myself	“Strongly disagree” (1) – “Strongly agree” (4)
6. All in all, I’m inclined to feel that I’m a failure	“Strongly agree” (1) – “Strongly disagree” (4)

Sense of coherence was measured using Antonovsky’s “Sense of coherence scale – Revised short version for children” [43, 46]. The Sense of coherence scale consists of three subscales: comprehensibility, manageability, and meaningfulness. For the present study, only the 4-item subscale measuring the sense of meaningfulness was used. According to Antonovsky this sub-scale is the most important of the three aspects and is a belief that things in life are interesting and a source of satisfaction, that things are really worthwhile, and that there is good reason or purpose to care about what happens [43]. The sense of coherence scale has been widely studied and has shown good validity across cultures [46]. The level of mastery was measured using Pearlin & Schooler’s Personal mastery 7-item scale, which has shown strong structural validity [44]. The self-esteem measure was derived from Rosenberg’s 10 item self-esteem scale [45]. Six items of the original 10 item scale were used. Turner et al. previously used this particular subscale for measuring self-esteem [47]. The exposure variables did not fulfill the assumptions of linearity, why the variables were categorized for analysis. The aim of the present study was to investigate the effect of different levels of psychological resources, why the exposure variables were categorized into tertiles at 33 and 66%, representing low, medium or high levels. The cut-off points for differentiating the tertiles are presented in Table 2.

**Table 2:** Cut-off points for categorizations of psychological resources

	2004			2007		
	Low	Medium	High	Low	Medium	High
Sense of coherence (4–20)	< 14	≥14 - ≤16	> 16	< 14	≥14 - < 16	≥16
Self-esteem (7–28)	< 18	≥18 - < 20	≥20	< 19	≥19 - < 21	≥21
Mastery (6–24)	< 20	≥20 - < 23	≥23	< 21	≥21 - < 24	≥24

### Covariates

As described in the background section socioeconomic measures, negative life events as well as health problems are possible confounders of the association between psychological resources and LMP [10–14], why the following covariates were chosen:

Independent variables included socioeconomic background defined by two perspectives: Equivalized household income and highest attained parental level of education. Equivalized household income was calculated from a yearly average of a 4-year period when the children were 7–10 years old, representing a period in the children’s lives where they were completely financially dependent on their parents. Equivalized income is a weighted average calculation divided by the number of household members based on a weight of 1.0 for the first adult, 0.5 for the second adult and persons above 14 years of age and lastly 0.3 for children below the age of 14 [48]. Income was recoded into tertiles corresponding to low (< 11,072 €), medium (11072–13,489 €), and high (> 13,489 €) yearly equivalized average income. Equivalized income average was only calculated if respondents did not miss more than 1 year of information regarding income. Highest attained parental level of education was obtained in year 2004 and categorized by primary level of education (< 10 years), secondary level (10–13 years) and tertiary level (> 13 years) of education. Information about socioeconomic factors was derived using information from the Central Office of Civil Registration (CPR) from the national registers in Statistics Denmark. In the register, the children are linked to their parents by their personal CPR-number [42]. These measures of socioeconomic factors have previously been validated [49, 50].

Further covariates in the analyses were: self-reported information about self-rated health, depressive symptoms and negative life events. Self-rated health was measured using a single question: “In general, how would you rate your health?” on a scale from 1 “poor” to 5 “excellent” health, derived from the SF-36 questionnaire [51]. The measure of self-rated health was dichotomized with (1–3) indicating moderate self-rated health and (4–5) indicating good self-rated health. Self-rated health was measured at age 15 and 18. Depressive symptoms were similarly measured at both ages using the 4-item version of Centre for Epidemiological Studies Depression Scale for Children (CES-DC) providing a score of 0–12. In analyses the CES-DC score was included as a continuous variable, with higher scores indicating a higher level of depressive symptoms [52]. Negative life events were measured at age 15 using 6 items from a 13-item scale of Newcomb et al. [53]. The scale and data of the measure of negative life events in the West Jutland Cohort study was previously described and analyzed in relation to LMP [10]. Three categories were made for analysis: 0 events, 1–2 events and 3–6 events.

### Statistical analysis

Baseline descriptive statistics of the study population in total and for males and females separately was performed and presented for all outcome, exposure and covariate variables as number and percentile distribution.

Binary multiple logistic regressions were used to study the associations between the exposure variables and outcome of LMP. This analytical approach was chosen to increase the interpretation of the results. All analyses were performed for the total population and stratified by gender. Covariates included in the analyses were chosen a priori based upon existing literature. The associations between psychological resources and LMP were analyzed in three different models.

First, a crude model examining the association of psychological resources and all covariates with LMP was performed independently. Secondly (model 1), independent analyses for the exposure variables of the psychological resources adjusting for the adolescents’ highest parental level of education and equivalized household income was performed. Lastly (model 2), the fully adjusted analysis was performed independently for each exposure variable adjusting for all additional covariates. All odds ratio estimates for (model 2) were presented with 95% confidence intervals with a significance level of *p* < 0.05. All calculations and analyses were carried out using STATA Statistical Data Package 15.1.

### Ethics

Use of the data was carried out under the same conditions and with the same purpose as originally collected. The study was approved by the Danish Data Protection Agency, according to Danish law for studies using questionnaire and register data (The Act on Processing of Personal Data - Act No. 429 of 31 May 2000). Written informed consent was not required in questionnaire or register-based projects.

## Results

Table 3 shows the characteristics of the final study population regarding exposure, outcome and covariates included in further analysis. From the final study population of 2982 participants, 2591 (87%) had a high level of LMP and 391 (13%) a low level of LMP. More females compared to males had a low LMP. In general, males reported having higher levels on all psychological resources, except for sense of coherence at age 15. The remaining covariates also demonstrated gender differences with males reporting the highest levels of self-rated health and the lowest levels of depressive symptoms and number of negative life events. No gender differences were seen regarding equivalized household income or highest parental education.

**Table 3:** Descriptive statistics of the study population (*n* = 2982)

	Age^a^	Total		Males		Females	
		n (%) or mean (SD)		n (%) or mean (SD)		n (%) or mean (SD)	
LMP 12 months	25–29	2982		1485		1497	
High		2591	(87)	1325	(89)	1266	(85)
Low		391	(13)	160	(11)	231	(15)
SOC	15	2967		1477		1490	
High		851	(29)	386	(26)	465	(31)
Medium		1138	(38)	581	(39)	557	(37)
Low		978	(33)	510	(35)	468	(31)
SOC	18	2149		986		1163	
High		686	(34)	313	(34)	402	(32)
Medium		725	(34)	340	(34)	388	(33)
Low		735	(32)	333	(32)	373	(35)
Mastery	15	2930		1468		1462	
High		936	(32)	513	(35)	423	(29)
Medium		1193	(40)	615	(42)	578	(40)
Low		801	(27)	340	(23)	461	(32)
Mastery	18	2088		953		1135	
High		658	(32)	318	(33)	340	(30)
Medium		734	(35)	349	(37)	385	(33)
Low		696	(33)	286	(30)	410	(36)
Self-esteem	15	2932		1466			
High		1234	(42)	736	(50)	498	(34)
Medium		975	(33)	502	(34)	473	(32)
Low		723	(25)	228	(16)	495	(34)
Self-esteem	18	2154		991		1163	
High		839	(39)	491	(50)	348	(43)
Medium		573	(27)	255	(26)	318	(27)
Low		742	(34)	245	(25)	497	(30)
Household income	7–10	2982		1485		1497	
High (< 13,489 €)		1082	(36)	548	(37)	534	(36)
Middle (11072–13,489 €)		999	(34)	498	(34)	501	(33)
Low (< 11,072 €)		901	(36)	439	(30)	462	(31)
Parents’ highest education		2875		1434		1441	
Primary (> 10 years)		358	(12)	177	(12)	181	(13)
Secondary (10–12 years)		1366	(48)	659	(46)	707	(49)
Tertiary (> 12 years)		1151	(40)	598	(42)	553	(38)
Depressive symptoms, mean (SD)	15	2.2	(2.2)	1.9	(1.9)	2.5	(2.4)
Depressive symptoms, mean (SD)	18	2.9	(2.3)	2.4	(2.1)	3.2	(2.4)
Self-rated health (SRH)	15	2963		1472		1491	
Good		2213	(75)	1178	(80)	1035	(69)
Poor		750	(25)	294	(20)	456	(31)
Self-rated health (SRH)	18	2147		984		1163	
Good		1383	(64)	714	(73)	669	(58)
Poor		764	(36)	270	(27)	494	(42)
Negative life events	15	2928		1468		1460	
No events		1772	(61)	918	(63)	854	(58)
1–2 events		1026	(35)	499	(34)	527	(36)
3–6 events		130	(4)	51	(3)	79	(5)

^*a*^*Age of respondents when data was collected; SD standard deviation*

Table 4 presents the analysis of psychological resources at the age of 15 and their impact on LMP in early adulthood. Overall, strong associations between all measures of psychological resources and LMP were observed in the crude models. The associations between psychological resources and LMP were all attenuated when adjusting for additional covariates. The association between mastery and LMP in the fully adjusted analysis (model 2) only remained strong for females with medium (OR: 1.9, 95% CI: 1.3–2.8) and high (OR: 1.6, 95% CI: 1.0–2.4) mastery compared to a low level of mastery. Furthermore, a high level of sense of coherence revealed a positive association with high LMP for females (OR: 1.6 95% CI: 1.0–2.4). Positive associations between mastery or self-esteem and LMP were present in model 1 for males but diminished when adjusting for all covariates.

**Table 4:** Psychological resources at age 15 and less than 12 months of passive labour market participation at age 25–29

	Total (***n*** = 2982)			Male (***n*** = 1485)			Females (***n*** = 1497)		
	Crude^**1**^	Model 1^a^	Model 2^**b**^	Crude^**1**^	Model 1^a^	Model 2^**b**^	Crude^**1**^	Model 1^a^	Model 2^**b**^
**Total**									
Sense of coherence									
Low	(ref.)	(ref.)	(ref.)	(ref.)	(ref.)	(ref.)	(ref.)	(ref.)	(ref.)
Medium	1.4 (1.1–1.8)	1.3 (1.0–1.7)	1.2 (0.9–1.5)	1.4 (0.9–2.0)	1.2 (0.8–1.8)	1.1 (0.7–1.7)	1.5 (1.1–2.0)	1.4 (1.0–2.0)	1.3 (0.9–1.9)
High	1.7 (1.3–2.2)	1.5 (1.1–2.0)	1.2 (0.9–1.7)	1.5 (0.9–2.2)	1.3 (0.9–2.1)	1.1 (0.6–1.7)	2.0 (1.4–2.8)	1.8 (1.2–2.6)	1.6 (1.0–2.4)
Mastery									
Low	(ref.)	(ref.)	(ref.)	(ref.)	(ref.)	(ref.)	(ref.)	(ref.)	(ref.)
Medium	1.8 (1.4–2.3)	1.9 (1.4–2.4)	1.6 (1.2–2.1)	1.3 (0.9–2.0)	1.4 (0.9–2.1)	1.6 (0.7–1.8)	2.1 (1.5–2.9)	2.2 (1.5–3.1)	1.9 (1.3–2.8)
High	2.1 (1.6–2.7)	1.9 (1.4–2.5)	1.4 (1.0–2.0)	1.7 (1.1–2.6)	1.6 (1.0–2.5)	1.2 (0.7–2.0)	2.1 (1.5–3.0)	1.9 (1.3–2.7)	1.6 (1.0–2.4)
Self-esteem									
Low	(ref.)	(ref.)	(ref.)	(ref.)	(ref.)	(ref.)	(ref.)	(ref.)	(ref.)
Medium	1.3 (1.0–1.7)	1.3 (1.0–1.7)	1.0 (0.8–1.4)	1.2 (0.8–1.9)	1.3 (0.8–2.0)	1.0 (0.6–1.6)	1.3 (0.9–1.8)	1.2 (0.9–1.7)	1.0 (0.7–1.5)
High	1.7 (1.3–2.2)	1.6 (1.2–2.1)	1.2 (0.9–1.7)	1.6 (1.0–2.6)	1.7 (1.0–2.7)	1.2 (0.7–2.1)	1.5 (1.1–2.1)	1.3 (0.9–1.9)	1.0 (0.7–1.6)
Equivalized household income									
Low (< 11,072 €)	(ref.)			(ref.)			(ref.)		
Medium (11,072–13,489 €)	1.1 (0.9–1.5)			1.3 (0.8–1.9)			1.0 (0.7–1.5)		
High (> 13,489 €)	1.6 (1.2–2.0)			1.7 (1.1–2.5)			1.5 (1.0–2.1)		
Parents highest education									
Primary (< 10 years)	(ref.)			(ref.)			(ref.)		
Secondary (10–12 years)	2.1 (1.6–2.9)			3.0 (1.9–4.7)			1.7 (1.1–2.5)		
Tertiary (> 12 years)	2.4 (1.8–3.3)			3.0 (1.9–4.7)			2.0 (1.3–3.1)		
Depressive symptoms, age 15	0.9 (0.9–0.9)			0.9 (0.8–0.9)			0.9 (0.9–1.0)		
Self-rated health, age 15									
Poor	(ref.)			(ref.)			(ref.)		
Good	1.7 (1.4–2.1)			1.7 (1.2–2.4)			1.6 (1.2–2.1)		
Negative life-events, age 15									
0 events	(ref.)								
1–2 events	0.6 (0.5–0.7)			0.5 (0.4–0.7)			0.6 (0.5–0.9)		
3–6 events	0.4 (0.2–0.6)			0.3 (0.1–0.5)			0.5 (0.3–0.9)		

^1^: crude estimates for all included variables

^a^: model 1: psychological resources (sense of coherence, mastery and self-esteem) individually adjusted for equivalized household income and parent’s highest education

^b^: model 2: psychological resources individually adjusted for all included covariates

^c^: confidence intervals (95%) presented for all models

Table 5 presents the analysis of psychological resources at the age of 18 and their impact on LMP in early adulthood. The crude analysis showed similar results as in Table 4 with overall strong associations between measures of psychological resources and LMP. Mastery presented positive associations with a high LMP for males at a medium (OR: 2.7, 95% CI: 1.5–3.8) and high (OR: 1.9, 95% CI: 1.1–3.5) level of mastery. Female mastery showed minor associations between medium or high mastery and high LMP compared to low mastery. A positive association between a high level of sense of coherence and LMP was present for the total study population in the fully adjusted analysis (OR: 1.5, 95% CI: 1.1–2.2), and when splitting on gender positive associations were still present for both males (OR: 1.7, 95% CI: 0.9–3.1) and females (OR: 1.4, 95% CI: 0.9–2.3).

**Table 5:** Psychological resources at age 18 and less than 12 months of passive labour market participation at age 25–29

	Total (***n*** = 2173)			Males (***n*** = 1000)			Females (***n*** = 1173)		
	Crude^**1**^	Model 1^a^	Model 2^**b**^	Crude^**1**^	Model 1^a^	Model 2^**b**^	Crude^**1**^	Model 1^a^	Model 2^**b**^
**Total**									
Sense of coherence									
Low	(ref.)	(ref.)	(ref.)	(ref.)	(ref.)	(ref.)	(ref.)	(ref.)	(ref.)
Medium	1.5 (1.1–2.0)	1.4 (1.0–1.8)	1.2 (0.8–1.6)	1.8 (1.1–2.8)	1.4 (0.9–2.3)	1.2 (0.7–1.9)	1.3 (0.9–1.9)	1.3 (0.9–1.9)	1.2 (0.8–1.8)
High	2.0 (1.4–2.7)	2.0 (1.4–2.8)	1.5 (1.1–2.2)	2.5 (1.5–4.2)	2.4 (1.4–4.1)	1.7 (0.9–3.1)	1.7 (1.1–2.6)	1.8 (1.1–2.7)	1.4 (0.9–2.3)
Mastery									
Low	(ref.)	(ref.)	(ref.)	(ref.)	(ref.)	(ref.)	(ref.)	(ref.)	(ref.)
Medium	2.4 (1.7–3.3)	2.3 (1.6–3.1)	1.9 (1.3–2.7)	3.2 (1.9–5.4)	3.1 (1.8–5.2)	2.7 (1.5–3.8)	1.9 (1.3–2.9)	1.8 (1.2–2.8)	1.5 (1.0–2.3)
High	2.4 (1.7–3.3)	2.2 (1.6–3.1)	1.7 (1.1–2.5)	2.9 (1.8–4.9)	2.5 (1.5–4.2)	1.9 (1.1–3.5)	2.0 (1.3–3.1)	2.0 (1.3–3.2)	1.5 (0.9–2.5)
Self-esteem									
Low	(ref.)	(ref.)	(ref.)	(ref.)	(ref.)	(ref.)	(ref.)	(ref.)	(ref.)
Medium	1.2 (0.9–1.6)	1.2 (0.9–1.6)	0.9 (0.6–1.3)	1.3 (0.8–2.1)	1.2 (0.7–2.1)	1.0 (0.6–1.8)	1.1 (0.8–1.7)	1.1 (0.7–1.7)	0.9 (0.6–1.4)
High	1.8 (1.3–2.4)	1.7 (1.2–2.3)	1.1 (0.8–1.6)	2.0 (1.2–3.2)	1.8 (1.1–2.9)	1.2 (0.7–2.1)	1.5 (1.0–2.3)	1.5 (1.0–2.3)	1.0 (0.7–1.7)
Equivalized household income									
Low (< 11,072 €)	(ref.)			(ref.)			(ref.)		
Medium (11,072–13,489 €)	1.0 (0.7–1.4)			1.3 (0.8–2.2)			0.9 (0.6–1.3)		
High (> 13,489 €)	1.4 (1.0–1.9)			1.6 (1.0–2.6)			1.3 (0.8–1.9)		
Parents highest education									
Primary (< 10 years)	(ref.)			(ref.)			(ref.)		
Secondary (10–12 years)	2.2 (1.5–3.2)			3.3 (1.8–5.8)			1.7 (1.0–2.7)		
Tertiary (> 12 years)	2.3 (1.6–3.3)			3.3 (1.8–5.8)			1.7 (1.0–2.9)		
Depressive symptoms, age 18	0.9 (0.8–0.9)			0.9 (0.8–1.0)			0.9 (0.8–0.9)		
Self-rated health, age 18									
Poor	(ref.)			(ref.)			(ref.)		
Good	1.9 (1.5–2.5)			2.3 (1.5–3.5)			1.7 (1.2–2.3)		
Negative life-events, age 15									
0 events	(ref.)			(ref.)			(ref.)		
1–2 events	0.6 (0.4–0.8)			0.4 (0.3–0.7)			0.7 (0.5–1.1)		
3–6 events	0.5 (0.3–0.9)			0.4 (0.2–1.1)			0.5 (0.3–1.1)		

^1^: crude estimates for all included variables

^a^: model 1: psychological resources (sense of coherence, mastery and self-esteem) individually adjusted for equivalized household income and parent’s highest education

^b^: model 2: psychological resources individually adjusted for all included covariates

^c^: confidence intervals (95%) presented for all models

Supplementary analyses were performed to investigate whether the changes in psychological resources between the age of 15 and 18 were associated with LMP in early adulthood. For this analysis respondents were categorized into four groups consisting of those who had a ‘low-low’, ‘high-low’, ‘low-high’, ‘high-high’ level of psychological resources at the ages of 15 and 18. All OR’s indicated higher odds of a positive LMP for respondents with a high level of SOC, mastery and self-esteem at both ages compared to those with a consistent low level of the respective psychological resources. Especially those with a ‘high-high’ level of mastery (OR: 2.1; 95% CI; 1.4–3.2) or a ‘high-high’ level of sense of coherence (OR: 1.5, 95% CI; 1.1–2.3) showed elevated odds of having high labour market participation at age 25–29 compared to those consistently staying at a low level of the psychological resources (see Additional file 1).

A sensitivity analysis was performed changing the value of categorizations of the exposure variables by +/− 1, which did not alter the results considerably. None of the odds ratios differed more than 0.2, with the highest alteration of 0.4.

Considering previous literature, the interactions between mastery and depression were tested. Furthermore, the interactions between all included psychological resources and gender were investigated considering the aim of the present study; however, all tested interactions were insignificant and did not contribute further to the models.

## Discussion

Overall, the results showed positive associations between high levels of psychological resources and a high level of LMP, with mastery and sense of coherence showing the strongest associations. Mastery and sense of coherence seemed to operate differently for both genders at different ages. Generally, the highest impact was seen for females at the age of 15 and for males at age 18. The supplementary analysis showed that those who stay at a high level of psychological resources seem to have increased odds of being more positively affiliated with the labour market in early adulthood when compared to those with a consistently low level.

The literature on the impact of self-esteem, sense of coherence and mastery in adolescence on later LMP is scarce. The present study found no substantial associations between self-esteem and LMP, which is in line with Orth et al. indicating that self-esteem does not predict trajectories of occupational status [26]. This however contradicts the results of Waddell [28] indicating that self-esteem displays a role in attaining employment 14-years after leaving high school. However, differences in study designs in relation to the construct and measurement method of LMP did compromise the comparability [28]. A study has presented significant gender differences in the development of self-esteem, where males in general have a higher level [22], which is somewhat supported by the results of the present study. A possible explanation to the contradicting results from previous literature could be that self-esteem may increase the odds of attaining a job in the initial job search but does not necessarily act as a protective factor for people disposed for longer periods of labour market exclusion. However, this argument still needs to be further investigated.

Previous research in a Danish context by Würtz et al. suggested that low sense of coherence in adolescent females (age 12–15) increase the odds of being on social benefits 12 years later [27]. Vastamäki indicated that high sense of coherence increased the chances of getting out of unemployment in a population below the age of 25 [24]. This is in line with the results of the present study indicating a moderate association between high sense of coherence and high LMP for females at age 15 and males at age 18. Danish and Swedish research, in general report age and gender differences in sense of coherence [46], with males reporting a higher level which similarly increases with age. This, together with previously mentioned research displays the knowledge gap in the impact of sense of coherence during adolescence on LMP and the potential differing effect by gender. The present study suggests that the impact of sense of coherence on males and females changes even during the few years of adolescence.

No studies investigating the impact of mastery during adolescence and LMP in early adulthood were found. Several studies have investigated mastery as a mediating factor between unemployment, economic hardship and depression showing strong associations with mastery as a mediating factor [31, 33]. In comparison, the present study showed that high levels of mastery in adolescence was strongly associated with a positive LMP in early adulthood, even when adjusting for the level of depressive symptoms. Pre-existing literature generally suggests looking at mastery as a mediating factor; however, with the results of the present study, perhaps mastery should also be viewed as a psychological resource promoting important life developmental stages such as entering of the labour market. Limitations of the present study are the use of subscales and the categorization of the exposure variables of psychological resources. Despite the strengths of using well-renowned and validated scales, no normal level of any of the psychological resources has yet been defined. Misclassification of exposure is therefore a potential risk. Additionally, the information of exposure was self-reported, inducing risk of information bias. Considering the prospective design, differential misclassification of exposure in relation to outcome is unlikely as data was collected at baseline (2004) and again in 2007, respectively 14 and 11 years before follow-up. However, there is a possibility that non-differential misclassification could potentially have induced over- or underestimations of the observed associations. Due to the long follow-up period of 14-years, a gap between data collections occurs where unknown factors to the present study could have biased the outcome. Another limitation of the study is that it was not possible to adjust for chronic illness or accidents among the participants since it could possibly have influenced the LMP. However, chronic illness or accidents would most likely be associated with the outcome and not the exposures and would therefore not lead to differential misclassification.

As previously presented, existing literature regarding LMP often observe LMP through different categorizations. Arguably, cutting high and low LMP at 12 months is a rough categorization and misclassification in relation to the outcome is therefore of high risk. However, as stated earlier the potential bias is expected to be non-differential. The decision of dichotomizing at 12 months was based on a desire to investigate those respondents who had been excluded from the labour market for longer periods of time. 6 months was deemed too low a cut-off point considering the age of the study population with many young adults completing education and therefore finding themselves in the transitional period between education and their first job. Supplementary analyses were performed using 6 months of LMP as outcome. The results supported the main findings of the present study, though the associations were weaker compared to 12 months of LMP.

The initial response rate of the West Jutland Cohort was high (83% in 2004), though no information on non-responders regarding exposure was available. An earlier study targeting the issues of non-participation in the West Jutland Cohort study identified that non-responders are more likely to come from homes with a lower educational and income level [40]. In the present study, it was identified, that LMP was strongly associated with the parents’ highest education and moderately associated with their equivalized household income. Selection bias is considered a risk if the association between the psychological resources and LMP would be different between respondents and non-respondents. However, given the rather large number of respondents in the study population and the broad contrast in the data of exposure variables, those from the lower socioeconomic classes included in the study are expected to be representative and not differ considerably from the non-respondents of lower socioeconomic classes. Selection bias due to non-participation is therefore not of high concern in the present study.

Major strengths of this study are the prospective design and vast questionnaire information providing possibilities of studying associations between psychological, physical and social factors combined with the Danish register information on important life outcomes. The prospective design allowed studying the direction of relationship between important life factors. The high initial response rate of 83% is considered a strength in combination with the use of register-based outcome measures providing complete information, which is considered having a strong validity and coverage of the Danish population [50]. The different data sources minimize the risks of bias in relation to misclassification and information problems. Furthermore, the availability of studying psychological factors at two different stages of adolescence is considered a strength, which provides deeper insight into the development of the changing impact of these exposures.

The external validity and generalizability of the present study is limited to young people in Denmark and other countries with similar welfare systems. Comparison to countries with different labour market models than Denmark should be made with caution. In a societal perspective the results of this study are essential in seeking to lower early labour market marginalization in order to minimize the long-term negative effects. The use of self-reported data opens the possibility of screening for low levels of psychological resources already during adolescence. The importance of early intervention is furthermore supported by the supplementary analyses of the present study, where maintaining higher levels of psychological resources through adolescence is important for LMP in early in adulthood. However, maintaining a positive level of psychological resources puts responsibility on social institutions in society. Governmental actions towards the educational sector such as funding actions to examine the health of students with more focus on these resources could provide opportunity of intervening, before the level of adolescents’ psychological resources declines. The educational sector seems the most ideal arena of promoting psychological resources, as the results of the present study indicates adolescents at ages 15–18 to be vulnerable. The present study has furtherly used formalized scales for measuring psychological resources, why it presents opportunity for measuring and monitoring resources in adolescents.

Considering mentioned limitations of the present study, such as the use of derivative scales for measuring exposures, future studies should be conducted using the full scales. Additionally, the relationship between the level of psychological resources throughout adolescence and later LMP should be investigated furtherly by use of more measure points.

## Conclusion

The study found that psychological resources of sense of coherence and mastery seem to act as protective factors of longer labour market exclusion during early adulthood. Self-esteem did not seem to be strongly associated with LMP in early adulthood. Substantial gender differences were observed, indicating that most of these psychological resources have different impacts depending on both age and gender. Moreover, maintaining psychological resources at a higher level throughout adolescence is important to increase the odds of a higher LMP in early adulthood.

Further research needs to be conducted focusing on studying how psychological resources operate for both genders at different ages in improving the future focus on psychological resources in childhood and adolescence.

## Supplementary information

**Additional file 1.** Psychological resource change scores between the age of 15 to 18 and the association with labour market participation.

## Data Availability

The datasets used and/or analysed during the current study are available from the authors on reasonable request.

## Abbreviation

LMP: Labour Market Participation
