# Supplementary material for: Psychological resources in adolescence and the association with labour market participation in early adulthood: a prospective cohort study
Source: BMC Public Health. 2020 Mar 24;20:386. doi: 10.1186/s12889-020-08531-w (PMC7092559; doi:10.1186/s12889-020-08531-w)
Supplement: Supplementary file 1 — Additional file 1. Psychological resource change scores between the age of 15 to 18 and the association with labour market participation. [file 12889_2020_8531_MOESM1_ESM.docx]

| **Additional file 1: Psychological resource change scores between the age of 15 to 18 and the association with labour market participation** | | | | | | |
| --- | --- | --- | --- | --- | --- | --- |
|  | **Total** |  | **Males** |  | **Females** |  |
|  | OR | 95% CI | OR | 95% CI | OR | 95% CI |
| Sense of coherence |  |  |  |  |  |  |
| 'low-low' | (ref.) |  | (ref.) |  | (ref.) |  |
| 'high-low' | 1.07 | (0.71 - 1.63) | 1.07 | (0.56 - 2.07) | 1.07 | (0.62 - 1.85) |
| 'low-high' | 1.20 | (0.76 - 1.91) | 1.19 | (0.59 - 2.41) | 1.14 | (0.62 - 2.12) |
| 'high-high' | 1.53 | (1.05 - 2.25) | 1.67 | (0.91 - 3.06) | 1.46 | (0.88 - 2.40) |
| Mastery |  |  |  |  |  |  |
| 'low-low' | (ref.) |  | (ref.) |  | (ref.) |  |
| 'high-low' | 0.97 | (0.64 - 1.46) | 0.63 | (0.31 - 1.29) | 1.22 | (0.72 - 2.08) |
| 'low-high' | 1.24 | (0.75 - 2.07) | 1.47 | (0.58 - 3.70) | 1.09 | (0.59 - 2.02) |
| 'high-high' | 2.07 | (1.36 - 3.15) | 1.89 | (0.92 - 3.89) | 2.05 | (1.21 - 3.47) |
| Self-esteem |  |  |  |  |  |  |
| 'low-low' | (ref.) |  | (ref.) |  | (ref.) |  |
| 'high-low' | 1.28 | (0.82 - 2.00) | 0.79 | (0.32 - 1.95) | 1.59 | (0.92 - 2.74) |
| 'low-high' | 1.22 | (0.71 - 2.10) | 0.87 | (0.29 - 2.60) | 1.30 | (0.68 - 2.48) |
| 'high-high' | 1.24 | (0.83 - 1.84) | 1.00 | (0.42 - 2.33) | 1.18 | (0.74 - 1.89) |

The analyses are adjusted for equivalated household income, highest parental education, depression (2007), self-rated health (2007), negative life-events (2004), CI: Confidence Interval; OR: Odds ratio.
